# The effect of BMI on the mid-term clinical outcomes of mobile-bearing unicompartmental knee arthroplasty

**DOI:** 10.1186/s12891-022-05001-9

**Published:** 2022-01-13

**Authors:** Yikai Liu, Huanshen Gao, Tao Li, Zian Zhang, Haining Zhang

**Affiliations:** 1 Department of Joint Surgery, the Affiliated Hospital of Qingdao University, Qingdao, 266000 Shandong Province China; 2 Department of Joint Surgery, Zaozhuang Municipal Hospital, Zaozhuang, 277000 China

**Keywords:** Arthroplasty, replacement, knee, Body mass index, Obesity

## Abstract

**Objective:**

To evaluate the impact of body mass index (BMI) on the mid-term clinical outcomes and survival in patients receiving a mobile-bearing unicompartmental knee arthroplasty (UKA).

**Methods:**

We retrospectively collected data from 355 patients who underwent UKA from June 2006 to June 2015, with a mean follow-up of 106.5 ± 22.5 months. Patients were assigned into four groups based on their BMI before surgery: normal weight group (BMI 18.5 ~ 22.9 kg/m^2^), overweight group (23 ~ 24.9 kg/m^2^), obesity group (25 ~ 29.9 kg/m^2^), and severe obesity group (≥ 30 kg/m^2^). The knee society score (KSS), knee society function score (KSFS), hospital for special surgery score (HSS), and range of motion (ROM) were assessed before the operation and at the last follow-up. The femorotibial angle (FTA) was assessed after the operation immediately and at the last follow-up. Kaplan–Meier survival analysis was performed among the four groups.

**Results:**

The KSS, KSFS, and HSS in all groups were markedly improved compared with the preoperative values (p<0.001), but the ROM score was not significantly different (p>0.05). There were significant differences in KSS (p<0.001) and HSS (*p* = 0.004) across the four BMI groups, and these differences were due to the severe obesity group. All groups exhibited an inclination of knee *varus deformity* at the last follow-up (*p* < 0.05). Moreover, no marked difference in the implant survival rate was found among the different groups (*p* = 0.248), or in the survival curves (*p* = 0.593).

**Conclusions:**

BMI does not influence the implant survival rate. The postoperative functional and quality-of-life scores were significantly improved in all groups. Obese (BMI ≥30 kg/m^2^) individuals should not be excluded from UKA.

## Introduction

Obesity plays a significant role in the occurrence and progression of knee osteoarthritis (OA) [1–3], and globally, obesity has been predicted to enhance the demand for knee replacement surgery [4–6]. Nevertheless, morbid obesity is traditionally considered a contraindication to knee replacement surgery due to low long-term implant survival. Unicompartmental knee arthroplasty (UKA) is a common therapeutic regimen for end-stage medial compartment OA. Even with many advantages over total knee arthroplasty (TKA), the revision rate of UKA is higher [7, 8]. Morbid obesity increases intraoperative complications including inadequate exposure and implant alignment, as well as postoperative complications such as tibial loosening, wound complications and respiratory complications [6–12]. Different survival results of fixed-bearing UKA have been reported, while the effects of high BMI on mobile-bearing UKA clinical outcomes are still unclear. In fact, several factors, such as small sample size, fewer revision cases, short-term follow-up, or simple binary analysis, have greatly limited the previous investigations studying the effect of BMI on the prognosis of UKA. Compared to fixed-bearing UKA, mobile-bearing UKA has less bearing abrasion but a higher bearing dislocation rate. Mobile-bearing prevents higher maximum peak pressures to concentrate on a small area and reduces bearing abrasion [13], but whether this change will benefit obese patients is unclear due to the possibility of increasing bearing dislocation-induced prosthesis revision [13]. We propose the hypothesis that a high BMI does not increase the revision rate, and is not a contraindication to mobile-bearing UKA. Here, we focused on the impact of BMI on mid-term clinical outcomes of UKA and performed a retrospective comparative study composed of 355 patients undergoing medial UKA.

## Materials and methods

A retrospective case note review consisting of several variables was performed for each individual, such as age at surgery, sex, weight, height, follow-up duration, and postoperative complications. The BMI of patients was determined using the following formula: weight/height [2]. According to the BMI classification standards of Asian adults defined by the World Health Organization (WHO) [14], the patients were assigned into four categories: normal body mass group (BMI 18.5–22.9 kg/m^2^, 33 cases with 38 knees), overweight group (BMI 23–24.9 kg/m^2^, 35 cases with 43 knees), obesity group (BMI 25–29.9 kg/m^2^, 97 cases with 110 knees), and severe obesity group (BMI ≥30 kg/m^2^, 28 cases with 38 knees). This study was approved by the ethics committee, and all patients signed informed consent forms.

### Surgical procedures

Oxford mobile-bearing UKA (Biomet, Inc.,Warsaw, IN) was used in operation. General anaesthesia combined with midthigh saphenous nerve block was applied to the patient. A tourniquet was used at the beginning of the operation. A paramedial incision is made from the superomedial edge of the patella to the medial border of the tibial tubercle. Bone resection was performed according to the order of cutting the tibial plateau, then the posterior condyle of the femur, milling the distal femur, and anti-impingement milling. The amount of osteotomy was measured before bone cutting. Minor adjustment of bone resection was made if the extension or flexion gap was not appropriate. The goal tibial slope is 7°, and this slope is built into the tibial guide. Finally, bone cement was applied, and the prosthesis was placed. The detailed procedures were performed according to Oxford experience [15].

### Outcomes

Preoperative and postoperative functional scores and imaging data were collected and recorded by an independent professional. Clinical outcomes were assessed using the knee society score (KSS), knee society function score (KSFS), hospital for special surgery score (HSS), and range of motion (ROM). In the radiological evaluation, the femorotibial angle (FTA) was evaluated based on the Oxford radiological criteria. Preoperative and postoperative FTA were determined according to the angle between the two lines drawn from the centres of the femur and tibia (over 175° was varus, 170°-175° was normal, and below 170° was valgus). A revision was defined as the removal, exchange, or addition of an implant component, including bearing exchange for bearing dislocation, or conversion to TKA.

### Statistical analysis

The statistical analysis was performed using SPSS 23.0 software for Windows, and the survival curve was made by GraphPad Prism 8.0. Statistical analyses were two-sided, and significance was set at *P* < 0.05. Preoperative and postoperative KSS, KSFS, HSS, FTA, and ROM were calculated, and ANOVA was used to compare these variables across various BMI groups. Paired t-tests were adopted to determine differences within BMI groups. The revision was evaluated using Kaplan–Meier survival analysis.

### Missing values

A total of 31 patients with 35 knees were lost to follow-up. Therefore, their revision status remained unknown.

## Results

### Cohort demographics

A total of 355 patients (408 knees) with medial compartment OA of the knee, who were treated with mobile-bearing UKA from June 2005 to June 2015, were selected as the research subjects in the present study. The postoperative mean follow-up was 106.5 ± 22.5 months (range 80 ~ 136 months). Of these patients, 31 patients (35 knees) were lost to the follow-up, and thus, 324 patients (373 knees) who met the inclusion criteria were ultimately enrolled in the present work. There were no marked differences in age (F = 1.392, *p* = 0.245), sex (χ^2^ = 3.347, *p* = 0.341), or follow-up time (F = 0.517, *p* = 0.671) among the four BMI groups, which indicated a similar patient characteristic of different BMI groups at baseline. (Table 1).

**Table 1:** Cohort demographics

BMI group	N patients (knees)	Mean age	Sex (M: F)	Mean follow-up
< 23	54 (64)	67.4 ± 9.0	17:37	119.6 ± 16.7
23- < 25	69 (81)	65.1 ± 8.2	25:44	114.3 ± 21.8
25- < 30	153 (172)	65.6 ± 7.2	50:103	115.8 ± 26.5
≥30	48 (56)	64.2 ± 10.2	10:38	116.2 ± 25.0
Test value		F = 1.392	χ^2^ = 3.347	F = 0.517
*P* value		0.245	0.341	0.671

### KSS and KSFS

The postoperative KSS and KSFS were dramatically improved in all groups compared with their corresponding preoperative values (Table 2; Table 3; *p* < 0.001), which indicated that UKA improved function to a large extent regardless of weight. Moreover, significantly different KSS at the last follow-up was found among the four groups (Table 2; *F* = 10.344, *p* < 0.001). Multiple comparisons were performed, revealing that the KSS in the severe obesity group was significantly lower than other three groups (Table 2; *p*<0.05), while the KSS did not significantly differ across the normal body mass group, overweight group and obesity group (Table 2; *p*>0.05), which implied that the KSS improvement has negative correlation with BMI.

**Table 2:** Comparison of the KSS among the four groups before the operation and at the last follow-up ($\overline{\text{x}}$ ± s)

BMI group	Preoperation	Last follow-up	Difference between last follow-up and preop	*T* value	*P* value
< 23	46.24 ± 12.65	89.15 ± 7.26	42.91 ± 10.27	30.70	<0.001
23- < 25	47.31 ± 14.17	87.21 ± 13.26	39.9 ± 15.14	21.89	<0.001
25- < 30	49.23 ± 13.93	90.37 ± 9.31	41.14 ± 13.70	37.14	<0.001
≥30	45.98 ± 11.88	80.05 ± 16.51	34.07 ± 16.36	14.43	<0.001
*F* value (between groups)	1.120	10.344			
*P* value (between groups)	0.341	<0.001

**Table 3:** Comparison of the KSFS among the four groups before the operation and at the last follow-up ($\overline{\text{x}}$  ± s)

BMI group	Preoperation	Last follow-up	Difference between last follow-up and preoperation	*T* value	*P* value
< 23	55.44 ± 13.06	89.83 ± 12.76	34.39 ± 15.59	16.21	<0.001
23- < 25	53.16 ± 10.48	87.47 ± 14.74	44.31 ± 20.77	17.72	<0.001
25- < 30	54.39 ± 20.27	85.01 ± 19.51	30.62 ± 21.28	17.80	<0.001
≥30	52.63 ± 16.11	82.28 ± 20.67	29.65 ± 19.61	10.48	<0.001
*F* value (between groups)	0.317	1.824			
*P* value (between groups)	0.813	0.143			

### HSS

The preoperative HSS was significantly improved in all groups compared with their postoperative values (Table 4; *p* < 0.001). Moreover, the largest absolute increase in reported HSS (Table 4; mean HSS improvement 30.66 ± 9.84) was found in the normal body mass group. In addition, significantly different HSS scores at the last follow-up were found among the four groups. (Table 4; F = 4.564, *p* = 0.004). Multiple comparisons were performed at the last follow-up, revealing that the HSS in the severe obesity group was significantly lower than other three groups (Table 4; *p*<0.05), while the HSS did not significantly differ across the normal body mass group, overweight group and obesity group (Table 4; *p*>0.05).

**Table 4:** Comparison of the HSS among the four groups before the operation and at the last follow-up ($\overline{\text{x}}$ ± s)

BMI group	Preop	Last follow-up	Difference between last follow-up and preop	*T* value	*P* value
< 23	60.61 ± 11.60	91.27 ± 10.37	30.66 ± 9.84	22.90	<0.001
23- < 25	62.49 ± 10.21	90.02 ± 11.16	27.53 ± 10.30	22.20	<0.001
25- < 30	61.36 ± 8.03	87.96 ± 10.87	26.60 ± 8.43	39.03	<0.001
≥30	59.63 ± 14.67	83.41 ± 14.90	23.78 ± 14.53	11.34	<0.001
*F* value (between groups)	0.787	4.564			
*P* value (between groups)	0.502	0.004			

### ROM

There was no significant improvement in ROM for each group (Table 5; *p* > 0.05). No significant difference in ROM was found among the four groups preoperatively or at the last follow-up (Table 5; *p* > 0.05), which indicated that BMI has no effect on the long-term ROM.

**Table 5:** Comparison of the ROM among the four groups before the operation and at the last follow-up (°, $\overline{\text{x}}$ ± s)

BMI group	Preop	Last follow-up	Difference between last follow-up and preop	*T* value	*P* value
< 23	123.3 ± 8.7	123.6 ± 7.5	0.3 ± 3.9	0.54	0.59
23- < 25	120.1 ± 9.7	121.0 ± 12.6	0.9 ± 7.3	0.95	0.35
25- < 30	121.9 ± 8.4	122.5 ± 10.1	0.6 ± 4.0	1.86	0.07
≥30	119.3 ± 12.1	119.7 ± 11.4	0.4 ± 5.3	0.52	0.60
*F* value (between groups)	2.107	1.542			
*P* value (between groups)	0.099	0.204			

### FTA

X-rays of the knee joint in the front and side positions and X-rays of the whole lower limb in the weight-bearing condition were performed for each inpatient to ensure the accurate measurement of FTA. The mean and standard deviation of immediately postoperative FTA was 173.5° ± 2.4°, while it was 174.9° ± 2.7° at the last follow-up. All groups were in the range of 170°-175°, except the severe obesity group at the last follow-up. Significantly different FTA was found among the four groups at the last follow-up rather than immediately postoperation (Table 6; *p*<0.05). Multiple comparisons showed that the FTA in the severely obese group was significantly higher than that in the other three groups (Table 6; *p*<0.05), which implied a slight varus tendency in the severely obese patients.

**Table 6:** Comparison of the FTA among the four groups between the immediate postoperation and at the last follow-up (°, $\overline{\text{x}}$ ± s)

BMI group	Last follow-up	Immediate postoperation	Difference between last follow-up and immediate postoperation	*T* value	*P* value
< 23	174.3 ± 2.7	173.4 ± 2.4	0.9 ± 2.3	2.88	0.006
23- < 25	174.8 ± 3.6	173.6 ± 2.7	1.2 ± 3.1	3.22	0.002
25- < 30	174.9 ± 2.4	173.3 ± 1.9	1.8 ± 2.9	7.25	<0.001
≥30	176.0 ± 2.9	174.2 ± 3.3	1.8 ± 2.7	4.62	<0.001
*F* value (between groups)	3.219	1.740			
*P* value (between groups)	0.023	0.159			

### Postoperative complications and prognosis

All patients in this study had no severe complications, such as venous thrombosis of the lower limbs, or cardiovascular and cerebrovascular accidents, and no cases of revision were caused by periprosthetic joint infection. Moreover, nine patients had unexplained abnormal joint noise and joint noise, while the symptoms gradually disappeared over time. There were seventeen revisions, including twelve cases due to bearing dislocation, three cases due to the progression of lateral compartment OA, and two cases for aseptic loosening of tibial components. No revision occurred in patients with a BMI of < 23 kg/m^2^. In addition, four cases of these revisions occurred in the group with a BMI of 18.5–22.9 kg/m^2^, representing 4.9% of UKA in this group. Nine (5.2% of the group) cases were found in the group with a BMI of 25–29.9 kg/m^2^, and four (7.1%) cases were found in the group with a BMI of ≥30 kg/m^2^. No correlation between indications for revision and BMI group was found.

**Fig. 1:**
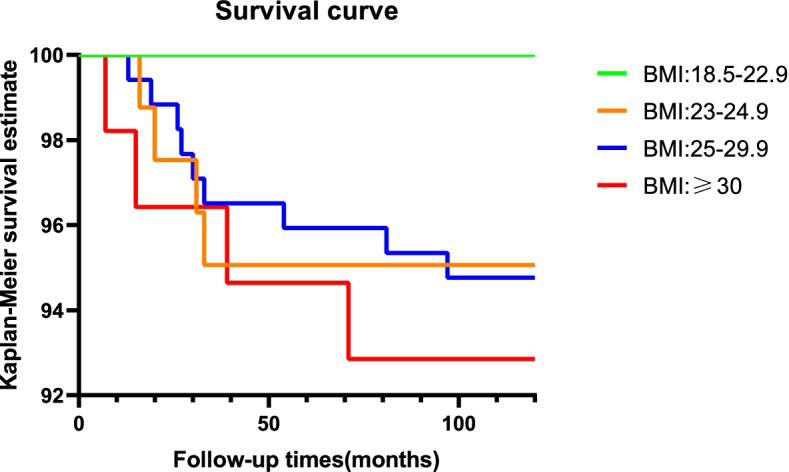
The revision was evaluated using Kaplan–MeierKaplan-Meier survival analysis. No difference in the prosthesis survival curve was found among the four groups. Prosthesis survival curves of the four BMI groups. No significant difference of prosthesis survival was found between the four BMI groups: 18.5–22.9, 23–24.9, 25–29.9, ≥30. (*p* = 0.593)

The survival rate of UKA for patients with a BMI of < 23 was 100%, and it was 95.06, 94.77, and 92.86% for the groups with a BMI between 23 and < 25, 25 and < 30 and ≥ 30, respectively (Table 7; Fig. 1). Moreover, no significant difference in implant survival rate was found among the various groups (χ2 = 4.124, *p* = 0.248) at the last follow up. No difference in the prosthesis survival curve was found. (*p* = 0.593).

**Table 7:** Survival rate of the implant in four groups

BMI group	N knees	Number of revision	Survival rate
< 23	64	0	100%
23- < 25	81	4	95.06%
25- < 30	172	9	94.77%
≥30	56	4	92.86%
Entire cohort	373	17	95.44%
At the last follow-up	χ^2^ = 4.124	P = 0.248	
Survival curve comparison		P = 0.593

## Discussion

The most important finding of our study was that both normal and high BMI patients showed acceptable clinical outcomes after UKA, even with significant differences of postoperative KSS and HSS compared with nonobese patients. Therefore, obesity should not be considered a contraindication of mobile-bearing UKA. For obese patients, mobile-bearing therapy is more strongly recommended than fixed-bearing therapy. Weight management is still beneficial for patients requiring UKA, as nonobese people have better functional scores in the long run.

Traditionally, obesity has been regarded as a contraindication in fixed-bearing UKA due to increased polyethylene wear and implant loosening [16]. Excessive weight or high BMI (BMI ≥ 30 kg/m^2^) will increase the wear of the components, thereby shortening prosthesis survival [17]. Moreover, the negative effects of obesity on UKA include excessive incision tension, delayed healing, higher infection rates, and lower activity levels in later rehabilitation training.

In this study, we assessed the effects of BMI on the mid-term clinical outcomes after UKA. With an average follow-up of 106.5 months, the postoperative KSS and HSS in the severely obese group rather than the postoperative KSFS, were significantly decreased compared with those in the other three groups, which indicated worse postoperative functional scores in obese individuals accepting UKA. The functional scores of different studies vary due to different follow-up times and different BMI classifications. Plate et al. [18] reported that patients had an average OKS of 34 at 6 months after surgery, which was not affected by BMI. Woo et al. [19] have shown that obesity does not affect the 2-year clinical outcomes of UKA. These studies indicated that the negative effect of obesity on the clinical outcomes of UKA may be latent after a short time. In addition, our findings were also supported by a previous work [20] showing that there was a significant difference in postoperative clinical outcomes between patients in the severe obesity group and those with a BMI of < 30 kg/m^2^, and the clinical scores in the severe obesity group were lower. Xu et al. [21] conducted a 10-year follow-up survey and reported that the KSFS and Oxford knee score (OKS) of patients with a BMI of ≥30 kg/m^2^ were significantly decreased at 10 years after surgery. However, a prospective study with a mean follow-up of 5.6 years conducted by Pandit et al. [22] showed that the clinical outcomes of 1000 mobile-bearing UKAs were similar between low weight patients and more than 82 kg patients. In a study with a minimum follow-up time of 7 years, Cavaignac et al. [23] found that there was no correlation between obesity and KSS. Plate et al. [18] suggested that BMI does not influence OKS, revision rates or readmission rates of robotic-assisted UKA in a mean follow-up of 34.6 ± 7.8 months. One reason that complicates the results is that multiple UKA prosthesis designs were used within single studies, for instance, fixed-bearing UKA and mobile-bearing UKA. Another reason is the differences in the BMI classification standards used by various studies for different races. The strength of our study is that only mobile-bearing UKA is involved, which may reduce the confounding bias caused by different prosthesis designs. In addition, the BMI classification used in this study was the ASIA standard defined by the WHO, which is suitable for Asians.

We did not find any significant difference in the mean age of UKA between the severely obese group and the other three groups, which contradicts the previous findings that obese patients are more prone to develop OA at a young age [24]. Gandhi et al. [24] have shown that BMI is a significant independent predictor of age at TKA. The mean age of patients with a BMI of ≥35 kg/m^2^ at the time of TKA was 8 years younger than that of individuals with a BMI of < 25 kg/m^2^. Indeed, we see young patients suffer from OA in outpatient clinics, usually we take osteotomy rather than UKA or TKA when the pain cannot be alleviated by drugs. The use of this operation may contribute to the different findings of our study.

We evaluated whether there was varus or valgus tendency in different BMI groups after UKA by comparing the FTA immediately after the surgery and the value at the last follow-up. The FTA results showed a tendency of slight varus over time, especially in the severely obese group, while it was still within the normal range in the other three groups. Obesity leads to increased joint load, uneven forces within the joints, and more serious damage to the articular cartilage, ultimately increasing *varus deformity* of the lower limbs, which was confirmed in our current research.

No difference in the survival of implants among different BMI groups, and, in particular, no trend towards decreasing survival was found with an increase in BMI. The most common failure of mobile-bearing UKA is bearing dislocations. Anti-impingement milling in our operations included posterior osteophyte removal and anterior flange of the femoral component cleaning, which may largely reduce impingement-induced bearing dislocation. We emphasized the stability and integrity of the medial collateral ligament (MCL), and during bone resection and milling, we used a Tesla retractor to protect the MCL. In addition to test extension and flexion gap balancing, we performed an anterior drawer test after surgery to ensure a suitable bearing and less possibility of bearing dislocation. Our findings were supported by Murray et al. [25], showing that the survival rate of mobile-bearing UKA did not decrease with increasing BMI in 2438 patients with a mean follow-up of 5 years. Kuipers et al. [26] showed that there was no reduction in implant survival after a mean follow-up of 2.6 years. Naal et al. [27] and Xing et al. [28] reported that the survival rate was similar between nonobese and obese patients receiving fixed-bearing UKA after a mean follow-up of 2 and 4.5 years, respectively [16].

Compared to mobile-bearing UKA, fixed-bearing UKA seems less stable and reliable. Several studies have performed fixed-bearing UKA in patients with high BMI and shown varying survival rates . Tabor et al. [29] have shown that obese patients have greater survival rates after a follow-up of 20 years. In contrast, in a cohort consisting of 67 knees with a mean follow-up of 3 years, Bonutti et al. [30] reported that patients with a BMI of > 35 kg/m^2^ have a higher risk of implant failure with a low survival rate of 88% compared with a survival rate of 100% in patients with a BMI of < 35 kg/m^2^. During flexion and extension movement, pressure tends to concentrate on a small area, which may increase bearing abrasion and prosthesis revision rate in fixed-bearing UKA. Different surgeons, dififferent follow-up times and other confounding factors are responsible for the diverse findings. Therefore, it is necessary to perform large comparative studies to evaluate the impact of obesity on the different types of UKA.

This study also has some limitations. First, this study was a retrospective study, which might cause possible selection bias, as patients were selected from a single hospital. A multicentre study could reduce such biases and allow a large number of patients to be studied. Second, the number of patients receiving revision was relatively small, so the evaluation of different possible risk factors including obesity that contribute to revision is hard to perform. Future studies with a larger sample size would facilitate a better interpretation of survival.

## Conclusions

In the present work, we showed that there was no difference in implant survival or indications for revision among patients with higher BMI. Moreover, we showed that the function and quality of life of all patients were remarkably improved after UKA. Although the severely obese (BMI ≥30 kg/m^2^) group showed lower postoperative functional scores than the other three groups, the improvement compared to preoperative functional scores was significant. Therefore, obese individuals (BMI ≥30 kg/m^2^) should not be excluded from UKA.

## Data Availability

The datasets generated during and/or analysed during the current study are available from the corresponding author on reasonable request.

## Abbreviations

OA: Osteoarthritis
UKA: Unicompartmental knee arthroplasty
TKA: Total knee arthroplasty
BMI: Body mass index
WHO: World Health Organization
KSS: Knee society score
KSFS: Knee society function score
HSS: Hospital for special surgery score
ROM: Range of motion
FTA: Femorotibial angle
ANOVA: Analysis of Variance
OKS: Oxford knee score
